# Identification and characterization of endophytic bacteria isolated from in vitro cultures of peach and pear rootstocks

**DOI:** 10.1007/s13205-016-0442-6

**Published:** 2016-06-03

**Authors:** Fakhra Liaqat, Rengin Eltem

**Affiliations:** 1 Department of Biotechnology, Graduate School of Natural and Applied Sciences, Ege University, İzmir, Turkey; 2 Department of Bioengineering, Ege University Faculty of Engineering, 35100 Bornova, Izmir Turkey

**Keywords:** Endophyte, Peach rootstock, Pear rootstock, Plant growth promoting properties, 16S rRNA gene sequencing

## Abstract

Endophytes are microorganisms which live symbiotically with almost all varieties of plant and in turn helping the plant in a number of ways. Instead of satisfactory surface sterilization approaches, repeatedly occurring bacterial growth on in vitro rootstock cultures of peach and pear was identified and isolated as endophytic bacteria in our present study. Five different isolates from peach rootstocks were molecularly identified by 16S rRNA gene sequencing as *Brevundimonas diminuta*, *Leifsonia shinshuensis, Sphingomonas parapaucimobilis Brevundimonas vesicularis*, *Agrobacterium tumefaciens* while two endophytic isolates of pear were identified as *Pseudoxanthomonas mexicana*, and *Stenotrophomonas rhizophilia*. Identified endophytes were also screened for their potential of plant growth promotion according to indoleacetic acid (IAA) production, nitrogen fixation, solubilization of phosphate and production of siderophore. All seven endophytic isolates have shown positive results for IAA, nitrogen fixation and phosphate solubilization tests. However, two out of seven isolates showed positive results for siderophore production. On the basis of these growth promoting competences, isolated endophytes can be presumed to have significant influence on the growth of host plants. Future studies required to determine the antimicrobial susceptibility profile and potential application of these isolates in biological control, microbial biofertilizers and degradative enzyme production.

## Introduction

Endophytes are bacteria or fungus which colonize healthy plant tissue, residing within a plant cell or between plant tissues with no apparent symptoms of disease (Nair and Padmavathy 2014). These endophytic microorganisms can spend their life cycle or a part of it while invading living tissue of the host plant deprived of producing any harm, with sometimes causing unapparent and asymptomatic infections (Kumala and Siswanto 2007). Endophytic bacteria frequently reside most of the plant and not a single plant species studied till date have been found free of endophytic bacteria (Nair and Padmavathy 2014). It is worth mentioning that, a huge number of existing plant species are host to more than one types of endophytes. Endophytic biology of all those plants is not fully studied. Therefore, it is a considerable opportunity to discover novel and valuable microorganisms among these plants (Muzzamal et al. 2011). While living symbiotically with the plants, these bacteria are known to stimulate plant growth by different ways including the production of phytohormones, solubilization of inorganic minerals like phosphate, fixation of atmospheric nitrogen and sequestration of iron. Additionally, they sometimes offer protection against pathogenic microorganisms and augmentation of ecological constraints such as drought, salinity and heavy metals (Khalifa et al. 2015). On the other hand, the presence of bacteria growth in in vitro plant tissue culture is generally declared as contaminants, which must be prohibited and eradicated (George et al. 2008; Abreu-Tarazi et al. 2010). Detecting the presence of endophytes in in vitro plant tissue culture and micropropagated plants is not extensively studied spot. Rarely, a few studies reported the manifestation of endophyte in in vitro plant tissue cultures (Almeida et al. 2009; Dias et al. 2009; Abreu-Tarazi et al. 2010; Moraes et al. 2012). The presence of valuable endophytic bacteria in plant tissue cultures and in their micropropagations may be more frequent than that reported (Abreu-Tarazi et al. 2010). In vitro plant tissue culture may offer a useful system and source to recover beneficial microorganisms resides within specific organs (Moraes et al. 2012). For a successful recovery of endophytes from plant tissues, it is foremost important to discriminate and eliminate any surface contaminants of plants, because endophytes purely reside inside the plant. In in vitro explant cultivation, plant materials are generally extensively surface sterilized, but inner flora of plant tissues cannot be avoided by surface sterilization approaches. The present study was, therefore, undertaken to identify and characterize endophytic bacteria appeared in in vitro explant cultures of GF677 (*Prunus amygdalus* × *P. persica*) peach rootstocks and OHF333 (*Pyrus communis* L.) pear rootstocks by culture-dependent technique; further, these isolates were also examined to explore the different characteristics predominantly plant growth promoting properties of endophytic isolates.

## Materials and methods

### Detection and isolation of endophytic bacteria from in vitro cultures of peach and pear rootstocks

In Plant Tissue Culture Laboratory of an agricultural company (Dikili Ciftlik in vitro Fidan A.S, Dikili-Izmir, Turkey), GF677 (*P. amygdalus* × *P. persica*) peach rootstocks and OHF333 (*P. communis* L.) pear rootstocks were routinely cultured on MS medium (Murashige and Skoog 1962) having sucrose (3 %) as key ingredient and agar (0.6 %), supplemented with benzylaminopurine (2.22 µM), indole 3 butyric acid (0.49 µM) and gibberellic acid 3 (0.29 µM). Cultures were incubated under a photoperiod of 16 h at 24 °C. It has been observed that in few batches of rootstock samples, instead of using several extensive surface sterilization methods prior to explant culturing, bacterial growth was repeatedly appearing within few days of cultivation. In contrast, no bacterial contamination was observed on control MS medium plates inoculated with the final wash solution of surface sterilization procedure which evidently proved the appropriateness of surface sterilization methods. Consequently, the bacteria frequently appeared on explant cultures were anticipated as endophytes and not the surface contaminates.

To further prove the occurrence of endophytic bacteria, explanation process was repeated using renowned surface-sterilized methodology for isolation of endophytes as described by Araújo et al. (2002). Rootstock samples were dipped in ethanol (70 %) for 1 min, then in sodium hypochlorite (2.5 %) for 20 min, subsequent washing with ethanol (70 %) for 30 s and finally rinsed three times with sterile distilled water. After surface disinfection, rootstocks were cut into pieces and were cultured on MS medium and incubated under same conditions. Plates were examined daily for bacterial colony development. Parallel to the samples again the final wash solution of surface sterilization procedure was also spread plated onto the MS medium plate which served as a control. Visible bacterial growth was isolated from the plates and aseptically streaked on nutrient agar plates and purified. Isolates were separated on the basis of their morphological characteristics, maintained in pure culture forms on the same medium and further processed for gram staining reactions as described by Hans Christian Gram (1884).

### Molecular identification of the isolates

16S rRNA gene sequences were used for molecular identification of isolated bacterial strains. Amplification of the 16S rRNA gene was performed with 27F and 1492R universal primers. Genes were amplified and sequenced by a commercial company with an ABI 3100 Genetic Analyzer (RefGen, METU, Technopark-Ankara, Turkey). The obtained sequence data were edited and aligned, using Geneious bioinformatics software (version 8.1) and a contiguous consensus sequence was generated. Aligned contiguous consensus sequence of 16S rRNA gene was used for homology search by the Basic Local Alignment Search Tool (BLAST) software (http://blast.ncbi.nlm.nih.gov) algorithm at National Center for Biotechnology Information (NCBI). The phylogenetic study of the 16S rRNA gene sequences of the isolates was conducted with Geneious version 9 beta using neighbor-joining method. Data obtained after sequencing have been submitted in the NCBI GenBank database to attain accession numbers.

### Study of plant growth promoting properties

#### Quantification of indoleacetic acid (IAA)

Quantitative detection of IAA was performed according to Acuña et al. (2011). Production of IAA was determined by colorimetric measurement at 530 nm using Salkowski’s reagent. Single bacterial colonies of each endophytic isolate were inoculated and grown under shaking at 120 rpm for 2 days at 30 °C in LB broth supplemented with tryptophan (1 mg/ml) as IAA precursor. After incubation, the cells were centrifuged at 3000 rpm for 10 min at 4 °C and 1 ml of supernatant was mixed with 2 ml of Salkowski’s reagent (150 ml of 95–98 % H_2_SO_4_, 7.5 ml of 0.5 M FeCl_3_·6H_2_O, and 250 ml distilled water) and incubated for 30 min at room temperature. The quantification of IAA was carried out using a standard curve with known concentrations of pure commercial IAA (Sigma-Aldrich, Co.). Uninoculated broth was used as negative controls and experiment is run in triplicate for each individual bacterium. Values are expressed in μg ml^−1.^

#### Determination of nitrogen fixation ability

To determine the isolates’ ability to fix atmospheric nitrogen, qualitative screening of growth was done on solid N-free medium (1 g K_2_HPO_4_, 5 mg FeSO_4_·7H_2_O, 1 g CaCO_3_, 0.2 g NaCl, 0.2 g MgSO_4_·7H2O, 5 mg NaMoO_4,_ 10 g glucose per litre, and 1.5 % agar at pH 7.0). Visible bacterial colonies on the N-free medium were used as the growth parameter and data were taken four and ten days post-inoculation (Ngamau et al. 2012).

#### Phosphate solubilization

The phosphate solubilizing activity of each of the isolates was determined by the method of Watanabe and Olsen (1965). Isolates were grown in 50 ml of NBRIP (National Botanical Research Institute’s phosphate) broth added with Ca_3_ (PO_4_)_2_ as insoluble forms of phosphate. Uninoculated NBRIP broth was used as control. The flasks were incubated on rotary shaker (180 rpm) at 30 °C for 7 days, after incubation broth was centrifuged at 10,000 rpm for 20 min. Supernatant was collected and autoclaved (121 °C for 20 min). Autoclaved samples were then filtered through 0.2 μm filter and were used for the determination of the soluble phosphate released into the solution. Optical density was taken at 700 nm and quantity of solubilized phosphate was measured with the help of KH_2_PO_4_ standard curve ranging up to 1 µg ml^−1^. Uninoculated broth was used as negative controls and experiment is run in triplicate for each individual bacterium. Values are expressed in μg ml^−1^.

#### Production of siderophore

Siderophore production characteristic of isolates was determined using CAS blue agar medium comprising chrome azurol S (CAS) and indicator hexadecyltrimethylammonium bromide (HDTMA) (Schwyn and Neilands 1987). CAS agar was prepared by supplementing sterilized MM9 salt medium (850 ml) comprising piperazine-N, N0-bis 2-ethanesulfonic acid (PIPES) (32.24 g), blue dye (100 ml), filter sterilized 10 % casaminoacid solution (30 ml) and 20 % glucose solution (10 ml). Isolates were inoculated on CAS agar plates and incubated at 28 °C and results were recorded after 24 h.

## Results and discussion

Rootstocks of peach and pear plants were observed to comprise endophytic bacteria appeared during in vitro cultivation on MS medium. Surface sterilization method for isolation of endophytic bacteria was found adequate as control plate has not shown any growth. Therefore, bacterial colonies appeared on sample plates can be well thought-out as endophytic bacteria of peach and pear rootstocks. To the best of our knowledge, there is no report found on the isolation of endophytes from peach and pear in vitro rootstock cultures. Though, there are few studies reported for the isolation of endophytic microorganisms from micropropagated plants (Almeida et al. 2009; Dias et al. 2009).

A total of seven morphologically distinct isolates were found as endophytes and all seven isolates were identified and characterized. Plant roots may contain other microbial diversity, but most of the endophytic microorganisms are unculturable and need culture-independent techniques to detect and identify. In this work, culture-dependent technique is applied; therefore, only those isolates which have shown growth under in vitro conditions were studied. Thus, all morphologically distinct isolates found were selected for characterization/identification.

Five morphologically distinct bacterial colonies were purified from peach rootstock samples and two different colonies from pear rootstock samples, bacterial isolates were given unique isolate numbers (Table 1) and processed for Gram-staining reaction. Five isolates from peach samples and two isolates from pear were found as Gram-negative rod shaped bacteria, while one isolate of peach was observed as Gram-positive short rods which were further molecularly identified using 16S rRNA gene partial sequencing. As 16S rRNA gene sequence offers precise identification of bacteria up to subspecies level, it is well thought-out as the most suitable method (Jill and Clarridge 2004). Using 16S rRNA gene sequence data, endophytes of pear rootstocks were identified as *Brevundimonas diminuta, Leifsonia shinshuensis*, *Sphingomonas parapaucimobilis*, *Brevundimonas vesicularis* and *Agrobacterium tumefaciens,* while endophytic isolates of pear rootstocks were identified as *Pseudoxanthomonas mexicana* and *Stenotrophomonas rhizophilia*. NCBI GenBank Accession number of all isolates and the 99 % similar isolate names are given in Table 1.

**Table 1:** 16S rRNA gene sequence analysis

Isolate number	Endophytic bacterial strain	Source plant	Gram staining reaction	NCBI accession number	NCBI database match	Percentage of identity
EGE-B-1	*Brevundimonas diminuta*	Peach	−	KP050788	*Brevundimonas diminuta* (EU430091)	99
EGE-B-2A	*Leifsonia shinshuensis*	Peach	+	KP050789	*Leifsonia shinshuensis* (KC345031)	99
EGE-B-2B	*Sphingomonas parapaucimobilis*	Peach	−	KP050790	*Sphingomonas parapaucimobilis* (AB680768)	99
EGE-B-3	*Pseudoxanthomonas mexicana*	Pear	−	KP050791	*Pseudoxanthomonas mexicana* (KF358268)	99
EGE-B-4	*Brevundimonas vesicularis*	Peach	−	KP050792	*Brevundimonas vesicularis* (KR085853)	99
EGE-B-5	*Agrobacterium tumefaciens*	Peach	−	KP050793	*Agrobacterium tumefaciens* (GQ181060)	99
EGE-B-6	*Stenotrophomonas rhizophilia*	Pear	−	KP050794	*Stenotrophomonas rhizophilia* (KM096602)	99

**Fig. 1:**
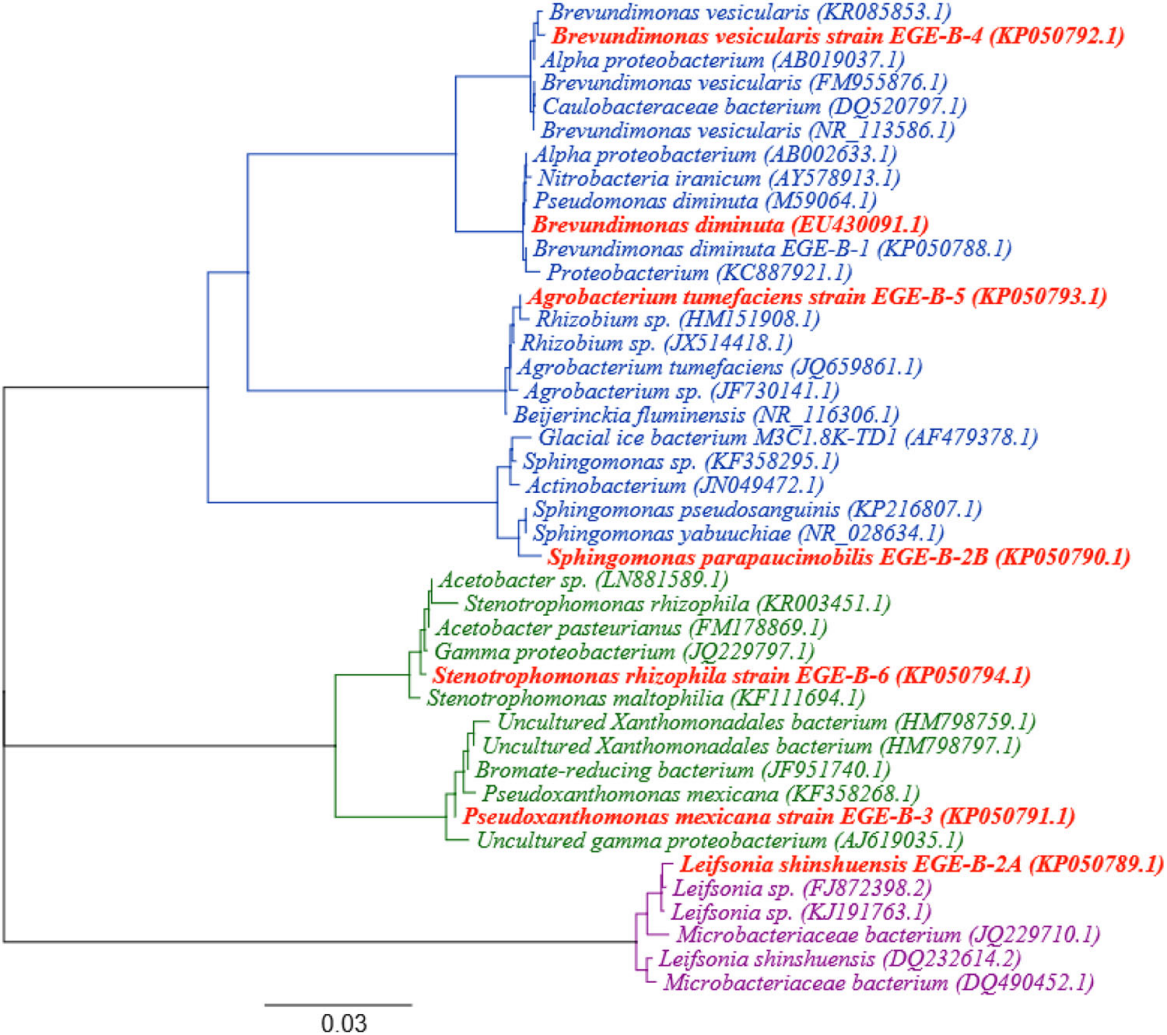
Phylogenetic analysis of 16S rRNA gene sequences of the bacterial isolates along with the reference sequences from NCBI. The analysis was conducted using neighbor-joining method. The scale bar represents 0.03 % substitutions of nucleotide

BLAST analysis of 16S rRNA gene sequence data provided us fairly precise grouping of bacterial isolates up to the species level. The phylogenetic studies of 16S rRNA gene sequence data of the our isolates plus the sequence data searched and retrieved from NCBI were performed with Geneious version 9 beta, applying Global Alignment type with free end gaps, 65 % similarity Cost matrix, Tamura Nei genetic distance model and neighbor-joining tree build method (Fig. 1). Out of total seven isolated endophytic bacteria six strains including *B. diminuta*, *P. mexicana, B. vesicularis*, *A. tumefaciens*, *S. rhizophilia* and *S. parapaucimobilis* belong to phylum Proteobacteria while one strain *L. shinshuensis* belongs to phylum Actinobacteria. Different genus of Proteobacteria phylum including *Brevundimonas*, *Sphingomonas* and *Pseudoxanthomonas* was previously reported as endophytes of seeds of cotton and cucumber (Hallmann et al. 1998), rice plant (Mano and Morisaki 2008), endophytic bacterial community of soybean root (Zhang et al. 2011), and endophytes of hybrid maize (Liu et al. 2012). Actinobacteria were also reported as endophytes of rice roots (Sun et al. 2008) and Taxus rhizosphere (Hao et al. 2008). Another report detected the occurrence of Actinobacteria, Alphaproteobacteria and Betaproteobacteria as endophytes in pineapple microplants (Abreu-Tarazi et al. 2010). These results can also be correlated with the results of other studies, which revealed Proteobacteria as a predominant plant growth promoting, pathogen antagonist and beneficial endophyte group (Andreote et al. 2006, 2009; Dias et al. 2009). From this comparative analysis, it can be determined that these species of endophytes isolated from peach and pear have a wide range of host specificity.

*Agrobacterium* species are well-known soil born phytopathogens and cause various plant diseases like crown gall disease. On the other hand, *Agrobacterium* genus is found among repeatedly isolated endophytic bacterial strains of root nodules of a variety of wild and cultivated legumes (De Lajudie et al. 1999; Wang et al. 2006; Saïdi et al. 2013). The taxonomy and classification of *Agrobacterium* are, however, questioned, and there are many disagreements regarding the nomenclature of *Agrobacterium* species as well as the genus. Most species have been controversially reclassified as *Rhizobium* species (Farrand et al. 2003; Chihaoui et al. 2015). Strains of *Agrobacterium* genus may be either tumorigenic, rhizogenic or non-pathogenic depending on the type of plasmids they comprise (Chihaoui et al. 2015). Numerous reports have revealed that *Agrobacterium* strains recovered from the root nodules have not shown ability to re-nodulate the original hosts, thus reported as nonsymbiotic (Wang et al. 2006; Liu et al. 2010). Some are also lacking in pathogenicity features (De Lajudie et al. 1999). Recently, several studies have declared *A. tumefaciens* as true plant endophytes with growth promoting abilities and have confirmed its occurrence and coexistence along with different endophyte species in diverse range of host plants (Procópio et al. 2009; Liu et al. 2010; Luo et al. 2011; Chen et al. 2012; Rashid et al. 2012; Mufti et al. 2015; Pandya et al. 2015). In our present study, *A. tumefaciens* is identified among the endophytes of in vitro peach rootstocks cultures and it has shown the best plant growth promoting properties as compared to other endophytes isolated.

### Plant growth promoting properties

Bacteria that inhabit the plant tissues as endophytes and which are not harmful for plant growth can display potentials to improve plant growth and can be advantageous for plant to augment symbiotic environment. The approaches by which the endophytes of peach and pear rootstocks could affect plant growth were examined by evaluating their capability for IAA production, nitrogen fixation, phosphate solubilization and siderophore production.

Endophytes can produce phytohormone IAA to promote plant growth (Mendes et al. 2007). IAA increases root size and distribution, resulting in a better nutrient uptake from the soil (Li et al. 2008). All the endophytic strains were checked for their ability to produce IAA by quantitative method and it was recorded that all the seven isolates gave positive results. Result was interpreted by comparing samples with positive (pure indoleacetic acid) and negative (uninoculated broth) controls. Quantities of IAA detected after comparing with standard curve of pure IAA are shown in Table 2. When screened for IAA production, the highest production rate was observed by strain *A. tumefaciens* (43.8 ± 2.3 µg ml^−1^) and lowest production was found in *B. vesicularis* (9.6 ± 2.9 µg ml^−1^). Numerous past studies have shown that growth promoting bacteria of different species produce IAA (Piccoli et al. 2011; Rana et al. 2011; Jha et al. 2012; Mufti et al. 2015; Yaish et al. 2015). Plant roots contain tryptophan which can be consumed by endophytic bacteria as a precursor for IAA production; therefore, IAA quantification is considered as common trait among the characterization of plant-associated bacteria.

**Table 2:** Growth promoting properties of isolated endophytic bacterial strains

Endophytic bacterial strains	Plant growth promoting properties			
	IAA Production (µg ml^−1^)	Phosphate solubilization (µg ml^−1^)	Nitrogen fixation	Siderophore production
*Brevundimonas diminuta*	32.8 ± 1.8	6.8 ± 1.1	+	−
*Leifsonia shinshuensis*	24.9 ± 1.7	20.4 ± 0.8	+	−
*Sphingomonas parapaucimobilis*	16.7 ± 1.2	9.8 ± 0.8	+	−
*Pseudoxanthomonas mexicana*	28.0 ± 0.9	15.8 ± 0.3	+	−
*Brevundimonas vesicularis*	9.6 ± 2.9	8.4 ± 1.3	+	−
*Agrobacterium tumefaciens*	43.8 ± 2.3	26.0 ± 1.1	+	+
*Stenotrophomonas rhizophilia*	10.8 ± 1.3	9.6 ± 1.6	+	+

Average values of three independent experiments for each isolate in triplicate ± standard deviation

Endophytic isolates were also screened for their nitrogen fixing abilities by growing these bacteria on nitrogen-free medium. It is found that all these isolates have shown growth on nitrogen-free medium which suggested their ability to fix atmospheric nitrogen (Table 2). Nitrogen-fixing ability of various endophytic bacteria was reported in many studies (Kuklinsky-Sobral et al. 2004; Sun et al. 2008; Li et al. 2010; Loaces et al. 2010; Pereira et al. 2012).

Phosphorus, is one of the plant growth limiting nutrients, which is added in the soil as fertilizer but usually becomes unavailable to the plant because of immobilization mechanism. However, bacteria living in plants play an important role in supplying phosphorus to plants. Endophytes are among those handy phosphate supplying bacteria (Wakelin et al. 2004). Phosphate solubilizing bacteria can change insoluble phosphates into soluble forms for plant through the process of acidification, chelation, exchange reactions, and production of organic acids (Chung et al. 2005). Endophytic bacteria in root zone are capable of increasing the availability of soil phosphorus to vegetation and improve plant growth (Duangpaenga et al. 2013). One of the most important phosphate solubilization mechanisms in plant-associated bacteria is the production of low-molecular weight organic acids which result in the acidification of the soil (Oteino et al. 2015). Isolates were screened quantitatively for their ability to solubilize phosphate. Results depicted that all isolates have ability to solubilize insoluble phosphate by producing phosphatase enzyme. Quantitatively phosphate solubilizing abilities of these bacteria range between 6.8 ± 1.1 µg ml^−1^ by *B. diminuta* to 26.0 ± 1.1 µg ml^−1^ by *A. tumefaciens* (Table 2). Previously, phosphate solubilizing abilities of endophytes have also been reported in several varieties of crops (Palaniappan et al. 2010). Likewise, Mufti et al. 2015 recently reported phosphate solubilizing capabilities of different endophytic isolates, including *A. tumefaciens* predominant among other endophytes with maximum phosphate solubilization activity.

The production of siderophores by endophytes is advantageous for plants, as it is one of the mechanisms to outcompete phytopathogens by inhibiting their growth within the plants (Sharma and Johri 2003). Siderophores produced by bacteria may promote the plant growth directly, by providing iron to plant, as iron accessibility to plants is usually low; therefore, organic chelators produced by bacteria will help in iron absorption or benefit plants indirectly, by obstructing the availability of iron to pathogens, thus restraining pathogen growth (Szilagyi-Zecchin et al. 2014).

Siderophore production properties were detected by chrome azurol S (CAS) agar. Out of seven endophytes, all isolates have shown growth on CAS agar but only *A. tumefaciens* and *S. rhizophilia* have shown positive results for siderophore production in the form of orange halo around the colonies (Table 2). Development of yellow to orange halo around the growth indicates iron chelation commenced by produced siderophores. Isolates showing no color change around growth were declared as negative for siderophore production property. Siderophore has the ability to take away the iron from the dye complex resulting in the color change to yellowish orange (Schwyn and Neilands 1987). These siderophores producing endophytes reduce the accessibility of iron for iron requiring phytopathogens by sequestrating available iron. Hence, they indirectly improve the plant growth (Alexander and Zeeberi 1991). Endophytic *A. tumefaciens* strain was recognized for its ability to produce siderophores in a past report by Luo et al. (2011) using CAS analytical method. There are many reports affirming the capacity of *Stenotrophomonas* species to produce different forms of siderophores with the help of universally applicable CAS assay. Chhibber et al. (2008) described ornibactin type siderophore production by *Stenotrophomonas maltophilia* and in another study Ryan et al. (2009) revealed its potential to produce compound enterobactin which is catechol type siderophore.

Current outcomes are consistent with the likelihood that individual biological roles can be mutually performed by different bacterial species of similar ecological environment. For example, IAA production, phosphate solubilization and nitrogen fixation properties were detected in all seven isolates, which were belonged to distinct species. Additionally, high score for IAA production also matches with phosphate solubilization. For example, the highest production of IAA and phosphate solubilization both were observed in *A. tumefaciens*. However, plant growth enhancement is an outcome of pooled potentials encompassed by multiple types of bacteria associated with these plants.

## Conclusion

In the end of our study, we do not claim the isolation and detection of every endophytic strain in peach and pear rootstocks. However, we concluded that some of the bacterial strains isolated from peach and pear have the ability to produce the growth regulator IAA, to fix nitrogen and to solubilize phosphate. Two of the strains also have the ability to produce siderophore. Five out of seven isolates belong to same phylum, thus we can relate such similarities to the endophytic ecology of bacterial species, and also to plant metabolism and nutrient accessibility. Further studies are required to reveal the potential of these endophytes as biofertilizers. Moreover, greenhouse and field investigations are recommended for confirmation of this potentiality. Antimicrobial susceptibility profiles of these endophytes could suggest the best options for their control in in vitro plant tissue culture.
